# Outcome of Intravenous Immunoglobulin-Transmitted HTLV-I, Hepatitis B, Hepatitis C, and HIV infections

**Published:** 2013-03

**Authors:** Mohsen Foroughipour, Farahzad Jabbari Azad, Reza Farid hosseini, Abbas Shirdel, Amir Reza Khalighi, Hadis Yousefzadeh, Homa Sadri, Toktam Moghiman, Hossein Hekmatkhah

**Affiliations:** 1 Neurology Department, Ghaem Hospital, School of Medicine, Mashhad University of Medical Sciences, Mashhad, Iran; 2 Allergy Research Centre, School of Medicine, Mashhad University of Medical Sciences, Mashhad, Iran; 3 Immunology Research Centre, School of Medicine, Mashhad University of Medical Sciences, Mashhad, Iran; 4 Internal Medicine Department, Ghaem Hospital, School of Medicine, Mashhad University of Medical Sciences, Mashhad, Iran; 5 Student Research Assembly of Mashhad University of Medical Sciences, Mashhad, Iran; 6 Preventive Cardiovascular Care Research Centre, Mashhad University of Medical Sciences, Mashhad, Iran

**Keywords:** Hepatitis B virus, Hepatitis C virus, Immune Deficiency, Intravenous Immunoglobulin

## Abstract

***Objective(s):*** Since each unit of Intravenous Immunoglobulin (IVIG) is obtained from different blood donors, blood-borne viral diseases is of high importance. We aimed at investigating the prevalence of various viral infections: Human T-cell Lymphotropic Virus Type 1 (HTLV-I), Hepatitis B (HBV), Hepatitis C (HCV), and Human Immunodeficiency Virus (HIV) among patients referred for IVIG therapy section in Mashhad University of Medical Sciences, Mashhad, Iran.

***Materials and Methods:*** A prospective study was conducted on 130 IVIG recipients admitted to different wards of our Medical Centre: Immunology, Hematology, and Neurology, in 2010. After filling the informed consent form, a 5 cc blood sample was initially taken from each patient. Viral infections including HTLV-I Ab, HIV-Ab, HBsAg, HBc-Ab, and HBV-Ab were assessed using the ELISA technique before and after six three months treatment.

***Results: ***Test results for HTLV-I Ab, HBsAg, HBc Ab, HIV Ab, and HCV Ab were negative in all cases before IVIG therapy. After receiving IVIG, two female cases with CIDP showed positive results for HBV Ab (0.8%) and HBS Ag (0.8%) with ELISA and only one patient confirmed with PCR. There was not any significant relation between HBV Ag (*P=*0.14) and HBC Ab with type of disorder (*P=*0.66).

***Conclusion:*** This study showed that HTLV-I viral replication and the other investigated viral transmissions do not occur in plasma; therefore, the IVIG products are safe.

## Introduction

Human T-Cell Lymphotropic virus type 1 (HTLV-I) is a member of Retroviridae family which has been associated with two main diseases: myelopathy/tropical spastic paraparesis (HAM/TSP) and adult T cell leukemia (ATL) (1). According to the previous reports, Mashhad, in the Northeast of Iran, has been suggested as an endemic area for HTLV-I infection since 1996 (2).

HTLV-I infected individuals remain life-long asymptomatic carriers. It has been previously established that HTLV-I replication is high in asymptomatic HTLV-I carriers (3) and that replication occurs in the blood (4, 5). Moreover, Cabral *et al* (6) claims that HTLV-I viral replication does occur in plasma. They recommend that other transmission pathways for HTLV-I should be investigated further. Intravenous Immunoglobulin (IVIG) is a highly purified antibody (mainly IgG) product prepared from blood and plasma donors for treatment of primary and secondary immune deficiencies (PIDs and SIDs) (7-9). It is also used for prophylaxis for infections in certain disease states and several numbers of autoimmune and systemic inflammatory diseases due to its immunomodulatory and pharmacodynamic mechanism (10-12). Variations in antibody levels of IVIG products are inevitable due to the production of IVIG from variable resource of human plasma (around 1000 samples required for each unit) (13). Since each unit of IVIG is obtained from many blood donors, blood-borne viral diseases would be a major issue of concern.

Screening against pathogens starts at the donation centres and the source plasma is tested against Hepatitis B (HBV), Hepatitis C (HCV), Human Immunodeficiency virus (HIV) and Human T-Cell Lymphotropic Viruses (HTLV-I).

During the past two decades the studies reported HCV transmission in PID patients that administrated Intravenous Immunoglobulin (IVIG) (14), HCV infection is an indolent infection in most healthy individuals (15, 16). 

 Moreover, repeated administration of IVIG antibody caused HBV and markedly prolonged incubation period of this disease in experimentally infected chimpanzees (17), whereas there are absolutely no documented reports of HIV and HTLV-I transmission among IVIG recipients. In this study, we aim to investigate the prevalence of various viral infections including HTLV-I, HBV, HCV, and HIV among patients referred for IVIG therapy to the Immunology, Hematology, and Neurology wards of Mashhad University of Medical Sciences, Mashhad, Iran.

## Materials and Methods

130 patients that received IVIG therapy in different wards of the Medical Centre i.e. Immunology, Hematology, and Neurology were enrolled in this study. This research project is approved by the Ethics Committee of Mashhad University of Medical Sciences (MUMS), which corresponds to the provisions of the Declaration of Helsinki in 1995. All patients’ documents were kept confidential. Study protocol was expressed for all patients and then the informed consent form was obtained. 

Five cc blood sample was initially taken from each patient. HTLV-I Ab, HIV Ab, HBsAg, HBc Ab, and HBV Ab were measured using the ELISA technique (IBL International GMBH, Hamburg, Germany). Similar immunological tests were repeated three months later. Patients with a negative test for HTLV-I Ab, HBs Ag, HCV-Ab, and HIV- who received IVIG were enrolled in this study. 

 The study^,^s studied exclusion criteria were: 1) Patients with a positive HTLV-I Ab, HBsAg, HCV Ab, and HIV test, 2) Patients receiving other blood preparations with the probability of viral transmission, 3) Cases with high risk factors for HIV, 4) Patients with a positive family history of HBV or HCV, 5) Cases with a sexual partner infected with HTLV-I, HBV, HCV, or HIV, and 6) Patients undergoing any kind of surgery or dental procedure three months before or after receiving IVIG therapy. The study variables included age, sex, serum HTLV-I Ab, HBsAg, HCV Ab, HIV Ab, HBc Ab levels, and type of the IVIG preparation.

The collected data was entered into SPSS software, version 11.05 (SPSS Inc., Chicago, IL, USA) for all statistical procedures. Differences in proportions of viral infections were analyzed by χ^2^ test or Mann-Whitney test for non parametric data. A two-tailed *P*-valve<0.05 was considered statistically significant. 

## Results

This study included 130 patients aged between 11 to 74 years with a mean age of 29.1±0.92 years old. The studied individuals were 76 (58.56%) female and 54 (41.5%) male. The most common diseases being treated by IVIG were Common Variable Immune Deficiency (CVID) (65.4%), Chronic Inflammatory Demyelinating Polyneuropathy (CIDP) (31.5%), and Guillain-Barre syndrome (3.1%).

HBsAg testing before IVIG injection showed negative results in all cases, whereas after receiving IVIG, it was reported positive and was confirmed by PCR only in one female patient (0.8%) who had CIDP. The test results for HBc Ab before IVIG therapy were all negative, but after receiving this type of treatment, it turned out to be positive in another female patient (0.08%) with CIDP. ELISA serologic test results for HTLV-I Ab, HCV Ab and HIV Ab before and after IVIG treatment were negative in all the studied cases. 

## Discussion

In this study, 130 patients under IVIG injections were studied for viral infection transmission after IVIG therapy. The results showed only one case of HBV transmission and another with HBC transmission. The previous study on IVIG recipients about anti-HIV antibodies reported the viral infection transmission in only one case, a female drug addict who belonged to a high risk group (AIDS). They concluded that IVIG recipients should not be regarded as a group at risk for AIDS (18).

Cabral *et al *(6) investigated the HTLV-I models to present two main mechanisms for replication: cell-to-cell and Tax-induced clonal expansion, detecting virions in plasma samples of asymptomatic carrier. For the first time ,they showed that among 26 plasma samples of the patients treated by DNAse enzyme to eliminate any DNA contamination before RNA extraction, two cases (8%) showed amplification for HTLV-I (*P=*0.05). However, our study showed that there was no HTLV-I viral replication in IVIG products.

Transmission of HCV via IVIG has been documented before (19-21). It was concluded that the severity and velocity of HCV infection in such patients was very high and it seemed to be related to the underlying cause for IVIG therapy (20).

However, in contrast to these studies, some reported high viral infections transmission of HBV and HCV. In a study by Yap *et al*, patients with non-A, non-B hepatitis following IVIG therapy were studied. They showed that 15 out of 17 (88%) cases that were initially negative for HCV-RNA turned in to positive after receiving IVIG therapy (22). Healey conducted a survey on the rate of HCV infection in acute hepatitis patients having received IVIG in England. It was reported that 82% of these patients were infected by this virus (23). 

In the present study, it is not clear whether the infection was transmitted through IVIG therapy or some other routes. Moreover, there was not any reported evidence on HIV and HCV transmission by plasma products. 

In the statistical analysis of the current study, no association was revealed between infection transmission and the type of disease being treated with IVIG. However, most common treated patients were CVIDs. Moreover, previous studies were in accordance with the results of the present study as IVIG recipients were especially those with primary immune-deficiency and those treated with IVIG after transplantation (24-27).

## Conclusion

This study showed that HTLV-I viral replication and other investigated viral transmissions do not occur in plasma, therefore, the IVIG products are safe. Furthermore, no relationship was found between viral infections especially HBSAg and HBC Ab among IVIG recipients. However examining the common viral antibodies through more sensitive methods, i.e., PCR, among serum donors is highly recommend. Moreover, the authors greatly recommend that plasma units not enter the IVIG production process unless they have been fully examined during a 3-6 month period. Thus, it seems that from the aspect of viral transmission, today’s IVIG is safe.

## References

[1] Gessain A, Barin F, Vernant JC, Gout O, Maurs L, Calender A (1985). Antibodies to human T-lymphotropic virus type-I in patients with tropical spastic paraparesis. Lancet.

[2] Rafatpanah H, Hedayati-Moghaddam MR, Fathimoghadam F, Bidkhori HR, Shamsian SK, Ahmadi S (2011). High prevalence of HTLV-I infection in Mashhad, Northeast Iran: a population-based seroepidemiology survey. J Clin Virol.

[3] Montanheiro PA, Oliveira AC, Posada-Vergara MP, Milagres AC, Tauil C, Marchiori PE (2005). Human T-cell lymphotropic virus type I (HTLV-I) proviral DNA viral load among asymptomatic patients and patients with HTLV-I-associated myelopathy/tropical spastic paraparesis. Braz J Med Biol Res.

[4] Best I, Adaui V, Verdonck K, Gonzalez E, Tipismana M, Clark D (2006). Proviral load and immune markers associated with human T-lymphotropic virus type 1 (HTLV-I)-associated myelopathy/tropical spastic paraparesis (HAM/TSP) in Peru. Clin Exp Immunol.

[5] Kira J, Koyanagi Y, Yamada T, Itoyama Y, Goto I, Yamamoto N (1991). Increased HTLV-I proviral DNA in HTLV-I-associated myelopathy: a quantitative polymerase chain reaction study. Ann Neurol.

[6] Cabral F, Arruda LB, de Araújo ML, Montanheiro P, Smid J, de Oliveira AC (2012). Detection of human T-cell lymphotropic virus type 1 in plasma samples. Virus Res.

[7] Gürcan HM, Ahmed AR (2007). Efficacy of various intravenous immunoglobulin therapy protocols in autoimmune and chronic inflammatory disorders. Ann Pharmacother.

[8] Bayry J, Lacroix-Desmazes S, Kazatchkine MD, Kaveri SV.Intravenous immunoglobulin for infectious diseases: Back to the pre-antibiotic, passive prophylaxis era? (2004). Trends Pharmacol Sci.

[9] de Gracia J, Vendrell M, Alvarez A, Pallisa E, Rodrigo MJ, de la Rosa D (2004). Immunoglobulin therapy to control lung damage in patients with common variable immunodeficiency. Int Immunopharmacol.

[10] Bayrakci B, Ersoy F, Sanal O, Kiliç S, Metin A, Tezcan I (2005). The efficacy of immunoglobulin replacement therapy in the long-term follow-up of the B-cell deficiencies (XLA, HIM, CVID). Turk J Pediatr.

[11] Ahmed AR, Dahl MV (2003). Consensus statement on the use of intravenous immunoglobulin therapy in the treatment of autoimmune mucocutaneous blistering diseases. Arch Dermatol.

[12] Masson PL (1993). Elimination of infectious antigens and increase of IgG catabolism as possible modes of action of IVIg. J Autoimmun.

[13] Lejtenyi D, Mazer B (2008). Consistency of protective antibody levels across lots of intravenous immunoglobulin preparations. J Allergy Clin Immunol.

[14] Razvi S, Schneider L, Jonas MM, Cunningham-Rundles C.Outcome of intravenous immunoglobulin-transmitted hepatitis C virus infection in primary immunodeficiency (2001). Clin Immunol.

[15] Allen HJ, Seeff L (2000). Recovery persistence and sequelae in hepatitis C virus infection: A perspective on long-term outcome. Semin Liver Dis.

[16] Thomas DL, Astemborski J, Rai RM, Anania FA, Schaeffer M, Galai N (2002). The natural history of hepatitis C virus infection: Host, visual and environmental factors. JAMA.

[17] Stephan W, Prince AM, Brotman B (1984). Modulation of hepatitis B infection by intravenous application of an immunoglobulin preparation that contains antibodies to hepatitis B e and core antigens but not to hepatitis B surface antigen. J Virol.

[18] Brémard-Oury C, Couroucé AM, Badillet M, Huchet J, Chapelle A, Bierling P (1987). Prevalence of anti-HIV antibodies in immunoglobulins recipients. Presse Med.

[19] Bjøro K, Frøland SS, Yun Z, Samdal HH, Haaland T (1994). Hepatitis C infection in patients with primary hypogammaglobulinemia after treatment with contaminatedimmune globulin. N Engl J Med.

[20] Bresee JS, Mast EE, Coleman PJ, Baron MJ, Schonberger LB, Alter MJ (1996). Hepatitis C virus infection associated with administration of intravenous immune globulinA cohort study. JAMA.

[21] Jonas MM, Baron MJ, Bresee JS, Schneider LC (1996). Clinical and virologic features of hepatitis C virus infection associated with intravenous immunoglobulin. Pediatrics.

[22] Yap PL, McOmish F, Webster AD, Hammarstrom L, Smith CI, Bjorkander J (1994). Hepatitis C virus transmission by intravenous immunoglobulin. J Hepatol.

[23] Sinclair J (2007). Intravenous Immunoglobulin (IVIG) Therapy – Practical Aspects. Starship Children’s Health Clinical Guideline.

[24] Healey CJ, Sabharwal NK, Daub J, Davidson F, Yap PL, Fleming KA (1996). Outbreak of acute hepatitis C following the use of anti-hepatitis C virus--screened intravenous immunoglobulin therapy. Gastroenterology.

[25] Rossi G, Tucci A, Cariani E, Ravaggi A, Rossini A, Radaeli E (1997). Outbreak of hepatitis C virus infection in patients with hematologic disorders treated with intravenous immunoglobulins: different prognosis according to the immune status. Blood.

[26] Bresee JS, Mast EE, Coleman PJ, Baron M, Schonberger L, Alter J (1996). Hepatitis C virus infection associated with administration of intravenous immune globulin. JAMA.

[27] Anonymous (1994). Outbreak of hepatitis C associated with intravenous immunoglobulin administration- United States, October 1993-June 1994. MMWR.

